# Next-Generation Potentiometric Sensors: A Review of Flexible and Wearable Technologies

**DOI:** 10.3390/bios15010051

**Published:** 2025-01-15

**Authors:** Mahmoud Abdelwahab Fathy, Philippe Bühlmann

**Affiliations:** 1 Department of Chemistry, University of Minnesota, 207 Pleasant St. SE, Minneapolis, MN 55455, USA; 2 Department of Chemistry, Faculty of Science, Ain Shams University, Abbasia, Cairo 11566, Egypt

**Keywords:** wearable sensors, potentiometry, sweat analysis, sport performance, clinical diagnosis, ionophore

## Abstract

In recent years, the field of wearable sensors has undergone significant evolution, emerging as a pivotal topic of research due to the capacity of such sensors to gather physiological data during various human activities. Transitioning from basic fitness trackers, these sensors are continuously being improved, with the ultimate objective to make compact, sophisticated, highly integrated, and adaptable multi-functional devices that seamlessly connect to clothing or the body, and continuously monitor bodily signals without impeding the wearer’s comfort or well-being. Potentiometric sensors, leveraging a range of different solid contact materials, have emerged as a preferred choice for wearable chemical or biological sensors. Nanomaterials play a pivotal role, offering unique properties, such as high conductivity and surface-to-volume ratios. This article provides a review of recent advancements in wearable potentiometric sensors utilizing various solid contacts, with a particular emphasis on nanomaterials. These sensors are employed for precise ion concentration determinations, notably sodium, potassium, calcium, magnesium, ammonium, and chloride, in human biological fluids. This review highlights two primary applications, that is, (1) the enhancement of athletic performance by continuous monitoring of ion levels in sweat to gauge the athlete’s health status, and (2) the facilitation of clinical diagnosis and preventive healthcare by monitoring the health status of patients, in particular to detect early signs of dehydration, fatigue, and muscle spasms.

## 1. Historical Overview

Ionophore-based potentiometry stands out as an appealing analytical technique owing to its high selectivity and straightforward instrumentation. Over the past sixty years, it has evolved into a well-established technique for routine analysis, with sensors developed for over 60 analytes. Widely employed across various domains, including clinical chemistry, environmental analysis, physiology, and process control [1,2,3,4,5], this method involves an indicator electrode known as the ion-selective electrode (ISE), which transduces the activity of a target ion into an electrical potential that serves as the measured signal in conjunction with a reference electrode. This setup typically operates under near-zero current conditions. Only a few examples of chronopotentiometry with ISEs, where a constant current is applied, have been reported [6,7,8,9].

Conventional ISEs, which contain a liquid contact to the inner reference electrode (often referred to as inner filling solution) have limitations that need to be addressed to meet the demands of many biological applications, such as cell- or tissue-level ion analysis and wearable sensors. Among the limitations of conventional ISEs are the evaporation of the inner filling solution, as well as fragility to both external pressure fluctuations and osmotic pressure caused by differences in the ionic strength of samples and the inner filling solution [10,11]. Consequently, for many applications, the transition from conventional ISE electrodes to solid-contact ISEs became imperative. Solid-contact ISEs, characterized by compact size, ease of maintenance, operational simplicity, and cost-effectiveness, may be seamlessly integrated with electronic controls as well as measurement and data acquisition units to acquire intricate biological and chemical information.

In 1970, Hirata et al. proposed a solid-state electrode for sensing Cu^2+^, utilizing a Pt wire coated with a Cu_2_S-impregnated silicone rubber sensing membrane [12]. Subsequently, in 1971, Cattrall and Freiser fabricated the first solid-contact electrode (devoid of an internal filling solution), referred to as “coated-wire electrode”, employing a Pt wire coated with a polymeric sensing membrane that was doped with a Ca^2+^ ionophore [13]. Freiser et al. subsequently extended the application of coated-wire electrodes to other ions [14]. While these coated-wire electrodes exhibited a Nernstian response towards their respective target ions, they suffered from significant potential drift, which can be attributed primarily to the formation of an aqueous layer between the metal/membrane interface, resulting in transmembrane ion fluxes and an unstable phase boundary potential at the interface of the sensing membrane and the underlying metal [15,16,17]. In 1985, Nicolskii and Materova outlined three conditions to enhance the potential stability of solid-contact electrodes: high exchange currents for the redox reaction that enables the transition between electronic and ionic conductivity, chemical equilibrium for this redox reaction (which requires that the direct and reverse electrode reactions occur at equal rates), and absence of side reactions that interfere with the main electrode reaction [18].

In an attempt to reduce potential drifts of these coated-wire electrodes, Cadogan and co-workers introduced in 1992 the use of a conducting polymer as intermediate layer (i.e., polypyrrole PPy) between the substrate and the ion-selective membrane [19]. This layer was designed to stabilize the measured potential by serving as an ion-to-electron transducer. The introduction of ISEs with a conducting polymer as solid contact marked a major shift in focus towards solid-contact ISEs, with numerous researchers exploring various materials as ion-to-electron transducers to achieve optimal potential stability. To date, conducting polymers and high-surface-area nanostructured materials are the most researched solid contact materials, which we will briefly review before focusing on recent advancements in all-solid-state wearable potentiometric sensors.

### 1.1. Solid-Contact Materials and Their Mechanism of Action

**Figure 1:**
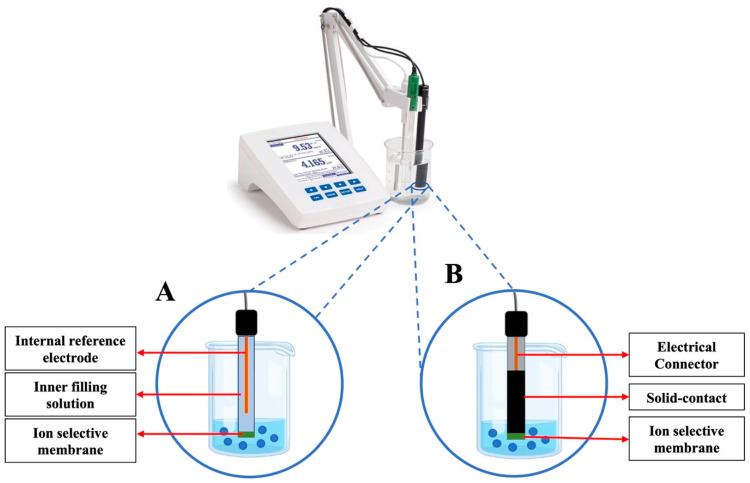
Illustration of structural differences between conventional ISEs (**A**) and solid-contact ISEs (**B**).

Solid-contact ion-to-electron transducer materials have garnered significant interest among researchers developing solid-contact ISEs as they are critical components needed to replace conventional ISEs, as depicted in Figure 1. Particularly for wearable sensors, the utilization of these materials is essential to enable portability, flexibility, and maintenance-free operation without compromising wearer comfort or well-being.

In the pursuit of improved solid-contact transducer materials, extensive efforts have been invested in identifying materials characterized by a high hydrophobicity, conductivity, capacity, and chemical stability. Notable advancements have led to the discovery of a range of well-performing solid-contact materials, including conductive polymers (CPs), carbon-based solid-contact materials, and various nanomaterials. The response mechanism is inherently tied to the type of solid-contact material employed and can broadly categorized into two types: (1) ion-to-electron transduction involving the redox capacitance of conducting polymers or molecular redox buffers and (2) ion-to-electron transduction featuring a high double layer capacitance, as in the case of high-surface-area carbon nanomaterials [20,21].

#### 1.1.1. Conducting Polymers (CPs) as Solid Contacts

Although polymers doped with electrolytes are ion conductors at temperatures above their glass transition temperature, (intrinsically) “conducting polymers” is a term usually used only for electron-conducting polymers with a contiguous backbone of sp^2^ hybridized atoms. Notably, many electron-conducting polymers exhibit higher conductivities when they are ion-doped, and under such circumstances, these conducting polymers exhibit both electron and ion conductivity.

Following the first successful application of polypyrrole (PPy) by Cadogan et al. in 1992 [19], conducting polymers were the primary solid material employed in solid-contact ISEs for over twenty years. ISEs with conducting polymers as solid contacts have demonstrated potential stability with a potential drift of 10 µV/h up to 8 days, reducing the need for recalibration [22]. Such performances provided much impetus to the field, prompting researchers to explore various other conductive polymers as solid contacts to achieve improved performances. Notable among these materials are polypyrrole (PPy) [19,23,24,25,26], poly(3-octylthiophene) (POT) [27,28,29,30,31,32], poly(3,4-dioctyloxythiophene) (PDOT) [33,34], poly(3-methylthiophene) (PMT) [35], polythiophene (PTh) [36,37,38], poly(α-naphthylamine) [39], polyaniline (PANI) [40,41,42,43,44,45], and poly(3,4-ethylenedioxythiophene) (PEDOT) [46,47,48,49,50,51,52,53,54,55,56,57,58,59,60,61,62,63]. These polymers differ in their ease of preparation, durability, hydrophilicity, cost-effectiveness, and electrical conductivity [64,65,66]. They can be deposited onto an electron-conducting substrate either through drop casting of polymer solutions or electrochemical polymerization (e.g., by chronocoulometry), making them suitable for mass production.

**Figure 2:**
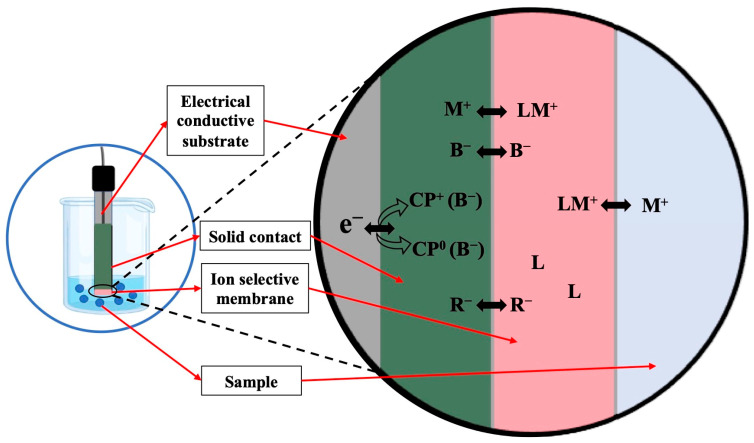
Schematic representation of a solid-contact ISE, featuring a cation (M^+^) selective membrane doped with ionophore (L) and anionic sites (R^−^) and a solid contact comprising a conducting polymer (CP) of high redox capacity, doped with the anion (B^−^).

Conducting polymers as solid-contact materials do not only need to display redox behavior but they also need to effectively adhere to the underlying electron conductor, which is typically a metal or carbon material. They function as ion-to-electron transducers through oxidation/reduction at the interface to the electron conductor (redox capacitance mechanism), as illustrated in Figure 2 for a cation-selective ISE. The mechanism of the redox capacitance mechanism depends on whether the anionic site, R^−^, comprised in the ion-selective membrane or the doping anion, B^−^ (e.g., Cl^−^), in the conducting polymer can transfer across the interface of the conducting polymer and the ion-selective membrane. Thus, the role of the conducting polymer can be represented as follows:

CP^+^ + B^−^_(SC)_ + L_(ISM)_ + M^+^_(aq)_ + e^−^_(C)_ ⇌ CP^0^_(SC)_ + B^−^_(ISM)_ + LM^+^_(ISM)_, (1)

CP^+^ + R^−^_(SC)_ + L_(ISM)_ + M^+^_(aq)_ + e^−^_(C)_ ⇌ CP^0^_(SC)_ + R^−^_(ISM)_ + LM^+^_(ISM)_. (2)

Here, C, SC, and ISM refer to the underlying conductor, solid-contact material, and the ion-selective membrane, respectively. CP^+^B^−^ represents the oxidized state of the conducting polymer acting as the solid contact, CP^0^ denotes the reduced state of the polymer, M^+^ represents the analyte ion (e.g., K^+^), and L and LM^+^ represent the ionophore and its complex with M^+^, respectively. Equivalent versions of Equations (1) and (2) can be formulated for anion-selective ISEs.

Equation (1) and (2) describe the overall reactions that involve equilibrium electron transfer at the interface of the underlying electron conductor and the conducting polymer, and equilibrium M^+^ ion transfer at the ISM/sample interface. The only difference between the two net reactions represented by Equations (1) and (2) is whether the anionic site, R^−^, or the doping anion, B^−^, transfer across the interface of the conducting polymer and the ion-selective membrane. Arguably, both processes may occur in parallel. If the ion transfer across this interface is kinetically very slow or thermodynamically unfavorable, the interface between the conducting polymer and the ion-selective membrane may also exhibit capacitance, in which case the response mechanism can be represented as follows:

CP^+^B^−^_(SC)_ + L_(ISM)_ + M^+^_(aq)_ + e^−^_(C)_ ⇌ CP^0^_(SC)_ + B^−^_(CP)_ + LM^+^_(ISM)_ (3)

Insufficient long term stabilities of the measured EMF of ISEs with solid contacts comprising conducting polymers have been explained by a number of factors. These include the reactivity of conducting polymers with redox-active species such as ambient oxygen, and the hygroscopic nature and light sensitivity of some conducting polymers [67,68]. Equations (1)–(3) also suggest that high activities of M^+^ in samples may promote the reduction in the conducting polymer when the potentiometer does not have a sufficiently high input impedance, or when the electrochemical cell permits leakage currents.

PANI was one of the conducting polymers used in the early development of solid-contact ISEs due to its high conductivity and the ease of its electrochemical synthesis. However, its hydrophilicity and sensitivity to pH present a significant challenge, particularly relating to long-term potential stability. The formation of a water layer between the conducting polymer and the ion-selective membrane interface, driven by PANI’s hydrophilicity, compromises the stability of these sensors. Despite attempts to mitigate pH sensitivity, including the approach by Liu et al. using a PANI/PMMA composite [69], and various efforts to enhance the analytical performance of PANI as a solid-contact transducer [70,71,72,73,74,75], problems such as the formation of a water layer at the interface to PANI and susceptibility to light and gas-induced side reactions cannot be avoided, limiting the overall reliability of PANI-based solid-contact ISEs.

To overcome these challenges, Gyurcsányi et al. enhanced the hydrophobicity of conducting polymers by doping PPy with a hydrophobic perfluorinated anion (PPy-PFOS), resulting in enhanced conductivity and improved reproducibility [76]. Lindfors et al. introduced a particularly hydrophobic counter anion, tetrakis(pentafluorophenyl)borate (TPFPhB^−^) as dopant for PEDOT, resulting in a K^+^ sensor with significantly reduced potential drift of 50 µV h^−1^ over 49 days [77]. Similarly, Lindner and colleagues utilized the same hydrophobic counter ion to dope PEDOT functionalized with a C_14_ alkane chain (PEDOT-C_14_) to improve interfacial hydrophobicity. This resulted in potential drifts of 0.02 ± 0.03 mV/day (n = 5) for a SC pH sensor and 0.1 ± 0.1 mV/day for a SC K^+^ ISE, without CO_2_ interference [78].

Lindner et al. also introduced the 7,7,8,8-tetracyanoquinodimethane (TCNQ/TCNQ^−^) redox couple as dopant for POT, resulting in a decrease in the potential drift of 0.1 mV/h [79]. Other doped conducting polymers have also been explored, including polyazulene [80,81], polypyrrole [82], nanocomposites of POT with molybdenum disulfide [83], POT blended with ruthenium dioxide [84], and various derivatives of PEDOT [85,86,87,88].

#### 1.1.2. Carbon-Based Materials as Solid Contacts

**Figure 3:**
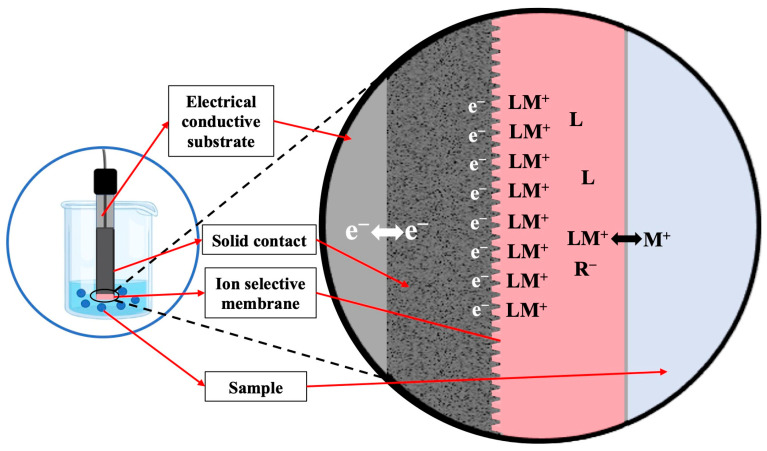
Schematic representation of a solid-contact ISE with a cation (M^+^) selective membrane doped with ionophore (L) and anionic sites (R^−^), as well as an electron-conducting carbon-based material as solid contact, exhibiting electrical double layer (EDL) capacitance at the interface of the carbon and the ion-selective membrane.

Carbon materials with high surface areas have recently emerged as promising alternatives and are increasingly integrated into wearable sensors. They address the limitations associated with conducting polymers, such as susceptibility to environmental factors (e.g., light and gas) and the formation of a water layer at the solid contact/ion selective membrane (SC/ISM) interface [89]. For this purpose, suitable carbon materials must exhibit both high electrical conductivity and a high degree of surface hydrophobicity. The latter prevents the formation of a water layer on their surface, reduces delamination during operation, and thereby prolongs their lifetime [13,90]. Solid contacts comprising carbon materials operate based on the electrical double-layer (EDL) capacitance mechanism, a transduction mode distinct from that for conducting polymers. It arises from the formation of an electrical double layer at the solid contact/ion-selective membrane interface, as illustrated in Figure 3.

As depicted in Figure 3, a carbon-based material with a high surface area, providing electron conductivity, is interposed as an intermediate layer between the electrically conductive substrate and an ion-selective membrane (depicted here for a cation, M^+^, as the target ion), serving as an ion-to-electron transducer through the formation of an electrical double layer at the solid contact/ion-selective membrane interface [91]. While ideal potentiometric measurements are currentless, any real potentiometer has a finite input impedance, resulting in a very small current through the ion-selective membrane. If the electrical current flows towards the voltmeter and no redox reactions are possible at the interface of the carbon material and the ion-selective membrane, this results in an accumulation of electrons on the carbon side of this interface and cations in the ion-selective membrane (i.e., for an ionophore-doped cation-selective electrode, ionophore complexes). If the current flows in the opposite direction, the carbon side of the interface charges up positively, and the membrane side of the interface accumulates anionic sites. The ratio of the separated charge across this interface and the resulting interfacial potential is the capacitance. For a high surface carbon material, this capacitance is very large, minimizing the phase boundary potential and, thereby, potential drifts over time, which explains the benefits of high-surface-area capacitive solid contacts [20].

Coated-wire electrodes operate using the same double-layer capacitance mechanism, with the metal serving as both the conducting substrate and solid contact. However, coated-wire electrodes suffer from two major drawbacks, i.e., high potential drift due to their small capacitance, as well as hysteresis effects as a result of the formation of water layers on the metal surface [18]. This illustrates the need for a high electrical double-layer capacitance resulting from a large interfacial surface area. This is best achieved with nano-structured materials rather than using outsized conventional capacitors.

Indeed, the use of carbon materials as solid-contact transducers has a long history. Already in 1971, Ruzicka et al. used cylindrical porous graphite rods fitted into a Teflon tube body and connected to a stainless steel wire for iodide sensing [92]. Also, in 1998, Chaniotakis et al. fabricated solid-contact ISEs comprising a conductive and porous activated charcoal matrix for K^+^ and NO_3_^−^ sensing [93]. However, these early endeavors did not prioritize the correlation between the contact area and the response characteristics, and the importance of achieving high surface hydrophobicity with minimal surface functional groups was not fully recognized.

In 2007, Bühlmann, Stein, and co-workers introduced solid-contact ISE with solid contacts made of three-dimensionally ordered macroporous (3DOM) carbon with uniform interconnected spherical pores of 420 nm, achieving drifts as low as 11.7 μV/h [94,95]. Further work with 3DOM carbon involving not only potentiometry but also characterization by electrochemical impedance spectroscopy (EIS), chronopotentiometry, and titrimetric quantification of surface functional groups, highlighted the importance of the high capacitance (2.1 F/g) and a high hydrophobicity of the carbon surface [96]. To improve the device-to-device reproducibility and facilitate fabrication, the same authors subsequently switched to colloid-imprinted mesoporous (CIM) carbon with 24 nm diameter interconnected mesopores [97,98]. This material showed high purity and hydrophobicity, along with low concentrations of redox-active surface functional groups, which led to enhanced potential stability and performance, particularly by resisting water layer formation and avoiding light, O_2_ and CO_2_ interference. The combination of CIM carbon with a redox couple as an internal reference gave a low standard deviation of *E*^0^ (as low as 0.7 mV) and a very low EMF drift (1.3 μV/h over 70 h).

Following the early work with 3DOM carbon, Crespo et al. introduced ISEs with single-walled carbon nanotubes (SWCNTs) as solid contacts [99], demonstrating a double layer capacitance of 60 ± 1 µF. Building on this, Bakker et al. further improved CNTs-based sensors by modifying multiwalled carbon nanotubes (f-MWCNTs) to increase their hydrophobicity [100]. This resulted in sensors with an emf drift of 0.04 mV/h and a double layer capacitance of about 1 mF, a performance comparable to that of traditional liquid-based systems.

CNTs have also been used as components of nanocomposite solid contacts. For example, Bobacka et al. used multi-walled carbon nanotubes (MWCNTs) doped into poly(3,4-ethylenedioxythiophene) (PEDOT) films to prepare K^+^ ISEs [101] with a redox capacitance of 721 µF, underscoring the effectiveness of CNT-based nanocomposites as solid-contacts for ISEs.

Similarly, Michalska et al. developed a nanocomposite of MWCNTs and poly(3-octylthiophene-2,5-diyl) (POT) as a transducer for ISEs [102]. In this case, POT functioned as both a dispersing agent and a stabilizer for the MWCNTs. An analysis using Raman spectroscopy showed that the transducer material was uniformly distributed across the sensor phase, preventing unwanted partitioning and ensuring the stability of the sensor. The MWCNTs–POT film displayed enhanced lipophilicity, with a water contact angle of 130°, and an increased capacitance of 0.2 mF compared to POT alone. These solid-contact ISEs exhibited an *E*^0^ reproducibility of less than 3 mV across six electrodes within a single batch.

Wang et al. used a nanocomposite of ordered bimetallic AuCu nanoparticles coupled with MWCNTs (oAuCuNPs-MWCNTs) as an intermediate layer between a gold-sputtered copper electrode and a PVC ion-selective membrane, effectively sensing Ca^2+^ and SO_4_^2−^ ions [103]. With a nanoparticle size (8 nm) and a high surface area, this resulted in capacitances of 54 µF and 105 µF for Ca^2+^ and SO_4_^2−^ ISEs, respectively. The nanocomposite provided good potential stability over 12 h (15 ± 3 µV/h for Ca^2+^ and 118 ± 16 µV/h for SO_4_^2−^ ISEs) and maintained its functionality for over 60 days, with no interference from light.

Recent research has further explored a range of carbon nanomaterials, such as reduced graphene oxide (rGO) [104,105], thiol functionalized reduced graphene oxide [106], graphene [107,108,109,110], and three-dimensional (3D) self-assembled porous graphene aerogels [111]. With their high surface area and capacitance, these materials offer good potential stability and reduced sensitivities to environmental factors compared to conducting polymers. Compared to conducting polymers, many solid contacts based on carbon materials exhibit enhanced resistance to water layer formation, oxidation, and light, making them suitable for harsh environments, even under elevated pressures of up to 100 bar [112,113]. However, despite their high conductivity and large capacitance, their interfacial potential is not always well defined. Additionally, fabrication challenges remain, as carbon nanomaterial-based films are typically insoluble in common solvents, complicating adhesion to underlying electron conductors. Though much of the recent work has focused on high surface area carbon materials with new geometries, the impetus of this work is often very exploratory in nature. More often than not, it is driven by the availability of a new type of carbon material, and not by specific features of the carbon that may be expected to improve the device’s performance. However, improvements in device performance, such as lower potential drifts and higher device-to-device reproducibility are more likely to arise in future from work that studies the interactions of these carbon materials with components of the sensing membranes at the molecular level and focusses on the chemical and mechanical long-term stability of these carbon materials [91,114].

#### 1.1.3. Other Solid-Contact Materials

In addition to conducting polymers and carbon-based materials, several other promising materials have been integrated as ion-to-electron transducers. The use of some of these materials provides ISEs with redox capacitance or EDL capacitance mechanisms, or both. Early studies by Bühlmann et al. introduced the concept of solid contacts stabilized by molecular lipophilic redox buffers by doping sensing membranes with Co(III) and Co(II) complexes of 1,10-phenanthroline ([Co(phen)_3_]^3+/2+^) paired with tetrakis(pentafluorophenyl)borate as a counterion [115]. This approach demonstrated an EMF standard deviation as low as 1.7 mV after 1 h, illustrating the potential of redox-active compounds to control the phase boundary potential and improve reproducibility by adjusting the ratio of [Co(phen)_3_]^3+^ and [Co(phen)_3_]^2+^. These findings set the stage for future developments in redox-active solid contacts.

Inspired by this approach, Michalska et al. replaced the redox buffer with a mixture of a reduced cobalt(II) porphyrin and an oxidized cobalt(III) corrole and combined that with MWCNTs (Ph(Co^2+^)/Cor(Co^3+^)–MWCNTs) [116]. Notably, the combination of Ph(Co^2+^) and Cor(Co^3+^) is not a redox buffer. Therefore, the resulting *E*^0^ reproducibility is poorly understood, although it is remarkably low, with 0.7 mV. In subsequent work [117], Michalska et al. found that a mixture of cobalt(II) porphyrin and a cobalt(III) corrole as the transducer material produced a reproducibility (*E*^0^) of 1.5 mV (n = 6) and a potential drift of 0.6 mV after 1 day. The presence of redox-active impurities is likely responsible for the strong performance observed, suggesting that better control over material composition could further improve both reproducibility and sensor longevity [118].

Mendecki and Mirica introduced conductive metal–organic frameworks (MOFs) as ion-to-electron transducers [119], which offer good electrical conductivity, structural tunability, and a large surface area. These MOF-based sensors demonstrated a capacitance of 204 μF and a potential drift as low as 11.1 μA/h, attributed to the hydrophobicity and stability of the MOFs at the electrode-membrane interface. Although redox-active, the MOFs presented so far do not exhibit redox buffer properties, which seems to explain why the potential drifts are not lower.

At the same time, Pięk et al. investigated molecular organic materials (MOMs) and their composites with carbon black in chloride-selective electrodes [120]. The mechanism of ion-to-electron transduction in these materials relies on two main phenomena: redox capacitance and electric double layer (EDL) capacitance. MOMs comprising TTF (tetrathiafulvalene) provide redox activity, enabling them to shuttle between oxidized and reduced states (TTF^+^/TTF), facilitating electron transfer between the ion-selective membrane and the electrode. Carbon black, on the other hand, increases the surface area and creates a porous structure, contributing to the formation of an EDL. This results in higher capacitance values, with carbon black–TTF^+^Cl^−^-modified electrodes achieving a capacitance of 2.8 mF and an impedance as low as 38.3 kΩ. Unfortunately, a long term potential drift under potentiometric conditions was not reported.

Criscuolo et al. demonstrated the potential of noble metal nanostructures, such as gold nanocorals and platinum nanoflowers, as solid contacts for lithium-ion selective electrodes [121]. These nanostructured materials provided an increase in capacitance as compared to flat metal electrodes by one to two orders of magnitude. The resulting sensors exhibited a potential drift of 30 µV/s. However, the high cost and complexity of synthesizing noble metal nanostructures may limit their widespread application unless drifts are further reduced.

Similarly, Cheong et al. developed ferrocyanide-doped redox-active screen-printed carbon electrodes for K^+^ sensing [122]. This system exhibited quite good *E*^0^ reproducibility, with standard deviations ranging from 0.7 to 3.6 mV between electrodes and stable potential readings (±2.8 mV) after conditioning for 6 to 27 h in deoxygenated solutions. This highlights the potential of ferrocyanide-doped systems for calibration-free measurements, although further testing in real-world conditions is necessary. Ferrocyanide-doped systems offer an intriguing balance between simplicity, cost-effectiveness, and performance, especially for portable or disposable sensor applications. However, their durability under practical conditions still needs validation.

Paczosa et al. investigated redox-active and electronically conducting ruthenium dioxide as a solid contact for K^+^ selective electrodes, with the hydrous form displaying superior performance [123]. Electrodes with hydrous RuO_2_ exhibited a higher capacitance (1.2 mF) and lower potential drift (1.5 µV/h) compared to the anhydrous counterparts, which had a capacitance of 188 μF.

More recently, Hassan et al. presented a novel approach for detecting trace levels of copper ions using manganese oxide (Mn_2_O_3_) nanoparticles dispersed in Nafion as a solid-contact material [124]. The screen-printed electrodes demonstrated significantly enhanced capacitance (91.5 μF) and minimized water layer formation. Furthermore, they introduced a paper-based potentiometric sensor for Pb(II) detection, employing vanadium pentoxide (V_2_O_5_) as the solid-contact material [125]. V_2_O_5_ showed high capacitance and effective ion-to-electron transduction through reversible redox processes, where electron transfer results in the oxidation of V^4+^ ions to V^5+^, thereby enhancing the overall capacitance. These advances highlight the potential of Mn_2_O_3_ and V_2_O_5_ as solid-contact materials for improving the performance and longevity of potentiometric sensors, especially for trace-level detection and miniaturized devices.

Shao et al. explored the use of two-dimensional MXene nanosheets (Ti_3_C_2_T_x_ and Ti_2_CT_x_) as solid-contact materials for calcium-selective electrodes [126]. Solid-contact ISEs with MXenes offered double-layer capacitances of 250 μF and 200 μF for the Ti_3_C_2_T_x_ and Ti_2_CT_x_ based electrodes, respectively. Unfortunately, the comment that the sensors exhibited “no significant potential drift” cannot be evaluated quantitatively.

### 1.2. Advancements and Future Directions

In conclusion, while substantial progress has been made in developing materials for SC-ISEs, challenges such as fabrication complexity, scalability, and long-term stability remain. Redox-active compounds are promising if they are used as redox buffers. Nanostructured materials like MXenes and noble metals are limited by cost and synthesis complexity and will only find applications if they perform better than other solid-contact materials, which is not the case to date. Moving forward, research efforts should focus on effective integration into practical, scalable, and long-lasting potentiometric sensors and the underlying causes of potential drift to achieve further improvements in drift performance for long-term calibration-free sensing.

## 2. Wearable Chemical Sensors and Substrate Design

Wearable sensor technology has experienced rapid growth in recent years, with its market value reaching approximately USD 32.63 billion last year, and the annual growth projected to exceed 15% by 2027 [127]. While commercial wearable sensors with the format of wristwatches already monitor physical activity through vital signs such as heart rate and blood oxygen, there is significant demand for more comprehensive wearable sensors to manage health conditions, prevent potential diseases, and enhance overall quality of life.

Wearable potentiometric sensors (WPSs) have emerged from an evolution of potentiometric technology, achieved through the integration of new materials and electronics to create portable and wearable sensors applicable for monitoring human body fluids without impeding user welfare. Several WPSs have been developed specifically for monitoring human health or sports performance [128,129,130], providing accurate measurements of vital signs in body fluids while avoiding the complications associated with traditional bioanalyzers, which often require blood draws and finger pricks.

The integration of potentiometric sensors into wearables must meet several key criteria: (i) high analytical and mechanical robustness during user activity, which is achieved through use of a solid-contact design, as discussed in the preceding sections; (ii) real-time health monitoring facilitated by wireless data transmission, enabling fully decentralized analysis [131]; (iii) ease of use, including effortless transport made possible by miniaturization; and (iv) affordability, which requires low manufacturing and data processing costs and enhances effectiveness and utility [132,133,134]. Challenges persist in manufacturing WPSs that meet all these criteria.

**Figure 4:**
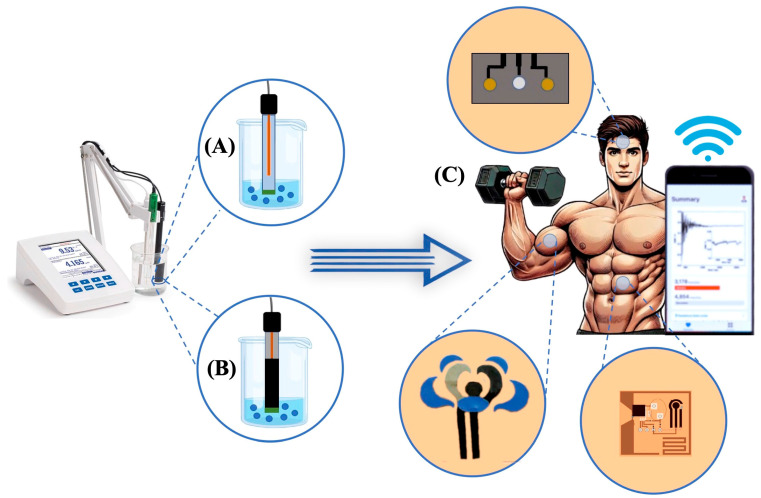
Evolution of ISEs from (**A**) a conventional design comprising liquid contacts to (**B**) the use of solid contacts for wearable sensing and (**C**) their application in the form of various designs of wearable potentiometric devices. Reproduced with permission from references [140,144,145].

The transition from conventional to solid-contact ISEs has resulted in various designs of wearable devices, as illustrated in Figure 4. These include epidermal patches (e.g., tattoos) [135,136], textiles [137,138,139], sweatbands [140,141], and eyeglasses [142,143]. These platforms are designed for convenience and enable non-invasive continuous monitoring of human fluids such as sweat, saliva, and tears for monitoring of patients in a clinical context and during daily activities of a diverse range of users, including athletes and the elderly.

The selection of the substrate material on which to build a WPS is a critical aspect of the design process, as it significantly influences the final cost, performance, lifespan, and comfortability. The substrate cannot be selected solely by consideration of the application type; other factors must also be considered, such as the nature of the conductive material to be deposited or coated onto the substrate, thermal and mechanical stability, substrate roughness, and surface energy [146]. Various substrates have been utilized in the manufacture of WPSs, ranging from commercially procured to homemade ones. Common substrates include ceramics, polyethylene terephthalate (PET), polydimethylsiloxane (PDMS), textiles, and paper. They can be categorized into two types: flexible and rigid substrates. Several manufacturing technologies can be employed to produce substrates for WPSs, including screen printing [147,148], gravure printing [149,150], stamp transfer [151], roll-to-roll [152], and inkjet printing [153]. Further details on recently reported all-solid-state wearable potentiometric sensors are provided in Section 3.

### 2.1. Non-Flexible Substrates

Non-flexible substrates, such as ceramic and metallic-based materials, are suitable for many applications due to their thermal stability, hardness, ability to accommodate various deposited conductive materials, and long lifespan [154,155,156]. However, their manufacturing process often involves sophisticated processing, leading to elevated costs. Consequently, they may not be the best choice for eco-friendly wearable potentiometric sensors, particularly those aiming for cost reduction. Additionally, their lack of flexibility can pose challenges, especially for sensors requiring adaptation to rugged surfaces.

### 2.2. Flexible Substrates

A range of flexible substrates are used in WPS fabrication due to their biocompatibility, high pressure tolerance, lightweight design, and moderate cost [157]. These features make them well-suited for real-time and portable use [158], and they are more compatible with mass production than many non-flexible substrates [159]. However, many flexible substrates have a shorter lifespan, and their smooth surface can present challenges for depositing or coating with conductive materials, potentially leading to early sensor failure.

#### 2.2.1. Paper Substrates

Paper, primarily composed of cellulose, stands out as an excellent flexible substrate for clinical and decentralized wearable potentiometric sensors. Its advantages include wide availability, low cost, flexibility, lightweight nature, and compatibility with biological samples [160]. Furthermore, paper-based sensors align with the concept of green electronics because their disposal is eco-friendly. The porous structure of paper substrates facilitates the application of conductive materials and recognition elements through physical adsorption and chemical coupling [161]. Conductive materials can be applied to paper substrates using inkjet printing, screen printing, or pencil drawing [162,163,164,165].

However, a notable challenge with paper-based sensors is their lack of water resistance, as demonstrated in a study by Cinti et al. [166]. This study compared different paper substrates and highlighted the effectiveness of wax paper in enhancing water resistance compared to regular paper. Bühlmann et al. [167] identified another critical limitation of paper-based chloride sensors: contamination of the paper substrate by chloride ions (Cl^−^) from device materials such as AgCl/Ag ink and ashless filter paper. This contamination can lead to an increased concentration of Cl^−^ in samples, adversely affecting the lower limits of detection (LODs) of these sensors. These findings emphasize the need for careful consideration of contaminants in the design of paper-based sensors.

#### 2.2.2. Polyethylene Terephthalate (PET)

Polyethylene terephthalate (PET) is a semi-crystalline polyester widely used as a substrate in various applications, including WPSs. PET substrates offer flexibility, durability, can withstand temperatures up to 220 °C [146], and are environmentally friendly to dispose of [168]. However, challenges arise from its hygroscopic [169] and hydrophilic nature, which can lead to the formation of aqueous thin layers between the substrate surface and conductive materials or ion-selective membranes, potentially reducing WPS lifespan. To address this problem, recent advancements have introduced solid-contact ion-selective and reference electrodes covalently attached to functionalized PET. This approach involves covalently bonding polyacrylate-based sensing and polymethacrylate-based reference membranes directly to surface-functionalized PET, effectively preventing delamination and enhancing the stability and longevity of the sensors [170]. PET substrates may still trap binders or solvents, particularly when coated with conductive carbon materials like graphite [171,172]. Compression rolling or photonic annealing may be used to remove trapped materials and improve the electrical conductivity of the graphite layer by enhancing its structure, alignment, and interface with the PET substrate [173].

#### 2.2.3. Polydimethylsiloxane (PDMS)

Polydimethylsiloxane (PDMS) belongs to the class of silicone polymers known as siloxanes. PDMS substrates offer flexibility and chemical inertness, making them suitable for manufacturing WPS. However, the chemically inert and hydrophobic nature of PDMS can hinder the adhesion of inks and conductive materials to its surface. To overcome this challenge, surface modification techniques, such as plasma treatment, are employed to enhance adhesion. For instance, Li et al. [174] utilized both oxygen and argon plasma treatments of PDMS substrates to improve the adhesion of silver inks containing epoxy resins, achieving comparable improvements with either plasma type.

Cheng et al. introduced a stretchable and highly functional ISE array that incorporates vertically aligned mushroom-like gold nanowires (v-AuNWs) embedded in a PDMS substrate [175]. This approach leverages v-AuNWs, fabricated using a solution-based seed-mediated method combined with photolithography, to create ultrathin, conformal devices. These v-AuNW-based sensors effectively address the limitations of traditional rigid ISEs by providing enhanced conformability and durability under strain. Equipped with specific ion-selective membranes, these sensors enable multiplexed and continuous potentiometric analysis of pH, Na^+^, and K^+^ in human sweat. They maintain high performance and stability even under 30% strain, and their integration with a flexible printed circuit board supports real-time, wireless on-body detection. The inherent stretchability and biocompatibility of gold nanowires make these sensors a promising solution for noninvasive and continuous health monitoring.

#### 2.2.4. Textiles

Textiles play a vital role in numerous human activities and have found applications in WPSs. They offer strengths, flexibility, and ductility, making them suitable substrates for WPS fabrication [176,177,178]. Synthetic fibers like polyamide (nylon) and polyester are commonly used due to their lower water absorption and better mechanical compatibility compared to natural fibers [179]. Textile substrates, whether fabrics, threads, or fiber surfaces, are often coated with conductive polymers to render them electrically conductive without altering their properties. Various methods, such as in situ polymerization, two-step polymerization, and chemical vapor deposition, are employed for coating [180,181,182,183,184]. Considerations must be made for washability and durability to maintain functionality and analytical performance. Numerous textile-based WPSs have been reported for monitoring human fluids [145,185,186,187], such as the detection of Na^+^ and K^+^ in human sweat [177].

### 2.3. Design of Wearable Potentiometric Devices

#### 2.3.1. Sweatbands

**Figure 5:**
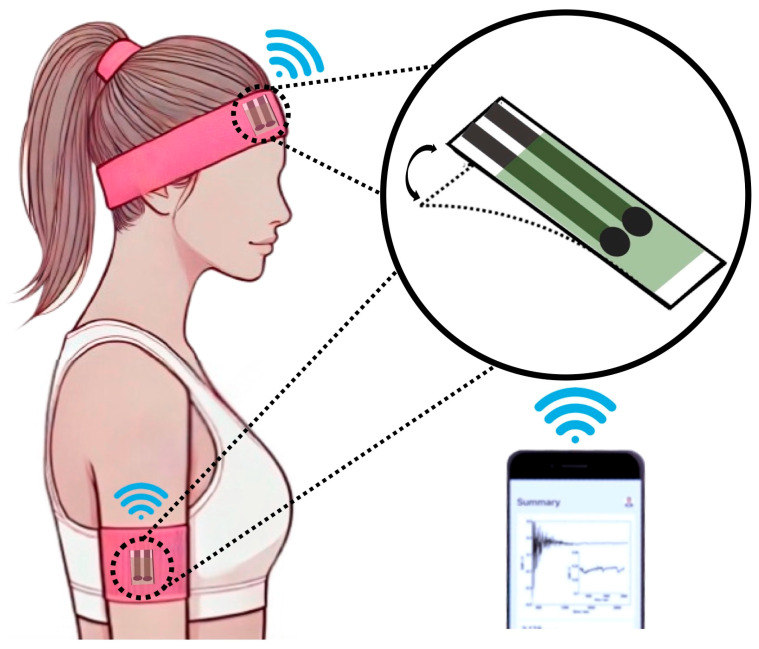
Schematic representation of sweatband-based WPSs for on-body measurements during physical activities. These devices typically consist of a sampling cell with reference and indicator electrodes, along with a wireless transmitter connected to a mobile phone serving as user interface for monitoring the target analyte during sports activities.

Sweatbands represent one of the most commonly used platforms for WPSs as they can be worn around various parts of the body, including the arms [188] and back [189], without compromising user comfort [190,191] (see Figure 5). Typically, potentiometric electrodes are integrated onto a flexible substrate in a separate process from the assembly of the sweatband itself. This means that the electrodes are first affixed to the flexible substrate, and this assembly can be performed independently of the final integration into the sweatband. This separation allows the electrodes to be replaced or disposed of without a need for specialized skills or extensive intervention. Once the substrate with the electrodes is attached to the sweatband and conforms to the body’s contours, calibration can be performed to enable accurate monitoring of the target analyte.

Diamond et al. [189] introduced the first WPS based on sweatbands for monitoring of Na^+^ levels in human sweat, utilizing a sampling cell based on absorbent materials employing capillary forces. Similarly, Javey et al. [192,193] developed a sweatband WPS using a sampling cell based on a water-absorbent thin rayon pad. These sweatbands were paired with an iontophoresis system to extract sweat from eccrine glands for effective monitoring of Na^+^ and Cl^−^ in cystic fibrosis patients [194]. However, this method sometimes caused discomfort or skin irritation due to burning, presenting a challenge in obtaining an adequate amount of sweat without affecting user comfort or causing adverse effects.

#### 2.3.2. Epidermal Patches

**Figure 6:**
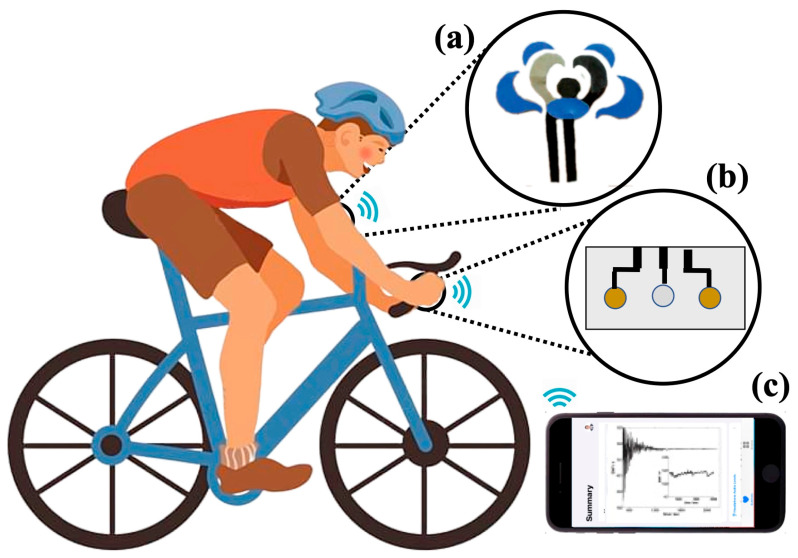
Schematic illustrating various designs of epidermal patch-based WPSs for on-body measurements during human activities: (**a**) tattoo-based WPS composed of two potentiometric electrodes (WE and RE); (**b**) electronic epidermal patch-based WPS with two WE for monitoring two analytes and an RE electrode; (**c**) user interface (e.g., mobile phone) serving as a wireless transmitter receiver. Reproduced with permission from references [140,144].

WPSs based on epidermal patches, which are thin, flexible devices designed to adhere to the skin’s surface, offer an ideal solution for achieving on-body adaptability, flexibility, and comfort [195]. These patches ensure optimal skin contact, particularly for monitoring human sweat, as depicted in Figure 6. Various designs of WPSs based on epidermal patches, including skin patches, tattoos, and bandages, have been explored to monitor targeted analytes in different physiological fluids on the human body [196,197,198]. To mitigate the potential side effects on the skin, such as toxicity, itching, and allergies [199,200], it is preferable to incorporate the sampling cell into the design, thus reducing cost and achieving full integration [201]. Fabrication of epidermal patch-based WPSs requires consideration of the flexibility and stretchability of the employed materials to mimic the skin’s physical performance during human activities and biocompatibility of the materials, especially in WPSs designed for wound monitoring [202].

Numerous studies have focused on designing tattoo-based WPSs [195]. For instance, Wang et al. developed a tattoo-based WPS using temporary, flexible, skin-compatible tattoo paper for direct epidermal measurements of perspiration [203]. Carbon ink reinforced with finely chopped carbon fibers was used as an ion-to-electron transducer. The Na-tattoo sensor exhibited a potential drift of 2.8 mV/h over a three-week period in the presence of the transducer material. The lack of sweat renewal in contact with the sensor, combined with contamination of the skin surface, affected the performance of these epidermal patch-based WPSs. To prevent direct contact between the electrodes and the skin, Wang’s group devised a microfluidic system worn on the skin for on-body measurements [204]. This system features micrometer-sized channel inlets that facilitate the collection and regeneration of sweat from sweat glands. The design ensures that the electrodes, integral to the sensing system, remain within the microfluidic channels, thus avoiding direct skin contact while allowing for accurate, real-time monitoring of Na^+^ and K^+^ levels in sweat. A conductive carbon ink, applied onto microfluidic gold electrodes, served as the solid-contact material, enhancing potential stability during measurements. The specific form of carbon in the ink was not explicitly reported in the original work. Conductive carbon inks typically contain finely dispersed carbon, such as graphite or carbon black, suspended in a fluid medium to provide high conductivity and mechanical stability.

Another approach to designing epidermal patch-based WPSs was introduced by Diamond et al. [205], who developed an electronic epidermal patch for monitoring Na^+^ and K^+^ in human sweat using a microfluidic system for sweat collection and storage. This system integrates flexible polyethylene terephthalate (PET) materials and poly(3,4-ethylenedioxythiophene) (PEDOT) as ion-to-electron transducers. The microfluidic design addresses key challenges in sweat sensing, such as reducing sensor lag and improving sensor lifetime by rapidly removing sweat from the sensing site. By incorporating a continuous flow mechanism, the system minimizes sweat accumulation, which can impact sensor stability and lead to erroneous readings due to sweat aging. The time to start on-body recording was 8 min, and the on-body test duration was 60 min. These results demonstrate the viability of electronic epidermal patch-based WPSs for continuous, non-invasive monitoring of physiological parameters, making them promising candidates for future wearable health technologies.

Xiu et al. developed a platform integrated directly onto a printed circuit board (PCB), enabling continuous monitoring of Na^+^ and K^+^ concentrations in sweat [206]. While solid-contact ISEs for Na^+^ and K^+^ monitoring have been described previously, the unique contribution of this work lies in the integration of the sensor with the PCB, which not only eliminates the need for external hardware but also simplifies the fabrication process and improves sensor stability through an electrochemical pre-treatment that enhances the extent of oxidation of the PEDOT/PSS layer. This resulted in a reduction in the relative standard deviation (RSD) of concentration measurement from 4.71% to 0.24% for Na^+^ and from 6.53% to 0.22% for K^+^. On-body tests indicate that the sensor maintains accuracy over 36 min of continuous use.

These studies demonstrate that while challenges related to sensor lifetime and stability remain, recent advancements—such as microfluidic integration—have significantly enhanced the capabilities of wearable sweat sensors. Addressing sensor lag and maintaining calibration accuracy in varying physiological conditions are critical for advancing these devices toward practical, long-term applications in health monitoring and fitness.

#### 2.3.3. Other Designs of WPSs

Various other designs have been developed for the on-body monitoring of human body fluids. For instance, Sempionatto et al. [142] introduced the first integrated wireless eyeglasses for monitoring of human sweat electrolytes and metabolites. The manufacturing process of this WPS relied on polyethylene terephthalate (PET) as a flexible substrate and incorporated conductive carbon ink as both conductive and solid-contact materials, applied with screen printing technology. It was connected to a wireless electronic board placed on the nose bridge of the glasses to obtain real-time measurements. Another innovative design was introduced by Miller et al. [207], who utilized a transdermal microneedle for monitoring K^+^ in interstitial fluid. This design combines a microneedle hollow core with a microfluidic chip to extract fluid through a channel towards solid-contact ISEs. Transducer materials such as 3D porous carbon and 3D porous graphene were employed. The 3D porous carbon-modified and graphene-modified electrodes exhibited membrane resistances on the order of 13 MΩ, but the latter demonstrated greater instability due to poorer membrane adhesion.

### 2.4. Design Progress and Outlook

Wearable potentiometric sensors have undergone remarkable advancements, evolving into innovative formats such as sweatbands, epidermal patches, and textiles. These designs, enabled by flexible and biocompatible substrates such as polyethylene terephthalate, paper, and polydimethylsiloxane, offer significant potential for non-invasive and continuous health monitoring. However, persistent challenges—such as short substrate lifespans, environmental sensitivity, and scalability—must be addressed to ensure reliable, affordable, and eco-friendly devices. While efforts in microfluidic integration, substrate surface modification, and wireless data transmission show great promise to improve the functionality of wearable sensors, the practical implementation on a larger scale remains a work in progress. Future advancements must prioritize durability and seamless integration while reducing manufacturing costs. These efforts will be crucial in transforming wearable sensors into indispensable tools for personalized healthcare and daily wellness monitoring.

## 3. Types of Biofluids and Wearable Potentiometric Sensor Applications

Conventional medical diagnosis of vital signs for early disease detection and continuous health monitoring is hindered by the inconvenience and inherent risks associated with blood drawing or finger pricks. Consequently, there is growing recognition of the importance of non-invasive medical diagnostic methods, particularly those based on the analysis of human vital fluids such as saliva, sweat, tears, and interstitial fluid. Wearable chemical sensors have emerged as a viable solution, with the added benefit that they do not require trained personnel for sample collection. Moreover, they provide user-friendly monitoring of personal health and facilitate early disease screening. In this section, we explore the characteristics of the different types of human biofluid of interest in this context, along with recent research findings centered on WPSs.

### 3.1. Sweat

Sweat contains a wide range of analytes, including ions, metabolites, and proteins, and has gained recognition for its potential to provide valuable information about an individual’s hydration status [208], physical stress, mineral loss [209], and certain diseases such as cystic fibrosis [210]. The pH of sweat typically ranges from 4.0 to 6.8 [211] and is influenced by factors such as weather conditions, stress levels, physical activity, and body temperature.

WPSs offer the ability to measure ions such as Na^+^, K^+^, Ca^2+^, Cl^−^, as well as metabolites like lactate, glucose, creatinine, and uric acid [212,213]. The utilization of WPSs for sweat analysis presents opportunities for early disease screening, personalized health monitoring, and performance optimization in athletes. However, sweat analysis poses challenges due to variations in the sweat secretion rate and sample evaporation [214]. To address these challenges, researchers have developed advanced sensor designs and microfluidic systems. These innovations include the integration of absorbent materials, microchannels, and sweat stimulation techniques aimed at improving sweat collection efficiency and minimizing sample loss (see Table 1 for a summary of recent research on all-solid-state WPSs for sweat analysis).

A notable trend in recent research is the further enhancement of the performance of WPS’s for sweat analysis, focusing on improving sensitivity, selectivity, and accuracy in detecting specific analytes. This trend aligns with the increasing demand for the reliable monitoring of biomarkers associated with various diseases and physiological conditions. The progress in WPS performance for sweat analysis can be attributed to advancements in sensor materials, fabrication techniques, and signal processing algorithms. One significant advancement is the integration of biocompatible materials into WPS’s for sweat analysis, which represents a pivotal development in health monitoring. The toxicity of ionophores and plasticizers used in ion-selective membranes (ISMs) poses a significant challenge due to the direct contact of the sensing membranes with the skin during tests [215]. Recently, Gan et al. developed a graphene oxide–poly(vinyl alcohol) (GO–PVA) hydrogel to coat the ion-selective membrane (ISM) of WPSs, aiming to improve the biocompatibility of these devices for sweat analysis [216]. This GO–PVA hydrogel coating did not interfere with the sensitivity and selectivity of K^+^ ISEs, while providing flexibility and reducing interference during real-time monitoring of sweat potassium ion levels. Although the study suggests the hydrogels may enhance the biocompatibility, further experimental evidence is needed to confirm their effectiveness in reducing leaching and toxicity.

Another significant trend is the integration of WPSs with wearable devices such as smartwatches, fitness trackers, or patches. This integration enables real-time monitoring and data transmission, allowing continuous tracking of sweat-related parameters. It provides valuable insights into an individual’s health status, facilitates immediate feedback, and enables the collection of long-term data for trend analysis and personalized health management.

To fully exploit the potential of sweat analysis using WPSs, several gaps and challenges must be addressed. Considerations regarding the long-term stability and reliability of WPSs for continuous sweat monitoring are crucial. Factors such as sensor drift, signal degradation, and biofouling can impact the accuracy and longevity of the sensor performance. For instance, potential stabilities reported for PEDOT-based sensors indicate drifts of 2.0–3.0 mV/h [192], while graphene-based sensors show drift as low as 0.18 mV/h [217]. Such drifts complicate the interpretation of time-variant signals, making it difficult to determine whether changes are due to sensor instability or genuine physiological fluctuations. Although repeatability is reported in many systems [194,205], validation of on-body measurements often remains incomplete. Additionally, most sensors exhibit on-body test durations ranging from 5 to 100 min, which is insufficient for many applications. More robust, long-term validation studies are essential to ensure the reliable, real-time operation of WPSs. Furthermore, standardization efforts must prioritize consistent sensor performance for accurate sweat analysis.

**Table 1:** Recent work on solid-contact WPSs for sweat analysis, highlighting key findings and technological advancements in sweat monitoring.

Substrate/Electrode Material	Transducer	ISM Polymer	Reference Electrode	Analyte	Working Range (mM)	Stability (mV h^−1^)	Lifetime (Week)	Type of Wearable	Electrode Fabrication	Physical Tests	Time to Start On-Body Recording (min)	On-Body Tests Duration (min)	Accuracy Validation	Wireless Output	Reference
PET/Au	PEDOT	PVC	PVB/NaCl	Na^+^/K^+^	[Na^+^], 20–120; [K^+^], 2–16	2.0–3.0	4.0	Sweat band/Head band	Photolithography	Bending	10	100	Yes	Bluetooth	[192]
PET/Au	PEDOT	PVC	PVB/NaCl	Na^+^/Cl^−^	10–160	-	-	Sweat band	Photolithography	-	20	25	-	Bluetooth	[194]
PET/carbon	PEDOT	PVC	liquid-junction RE	Na^+^	0.01–100	2.4 ± 0.6 over 4 h	-	Sweat band	Screen printing	-	20	70	-	RFID	[188]
PMMA/carbon	PEDOT	PVC	HMIM-FAP/PMMA-*co*-BMA/DEHP	Na^+^	0.1–100	-	-	Wrist band	Screen printing	-	8	60	-	Bluetooth	[191]
PET/carbon	PEDOT	PVC	HMIM-FAP/PMMA-*co*-BMA/DEHP	Na^+^/K^+^	0.1–100	-	-	Epidermal patch	Screen printing	-	8	60	-	Bluetooth	[205]
PET/Ag	PEDOT	PVC	PVB/NaCl	Ca^2+^	0.25–2	1.1 over 4 h	-	Sweat band	Photolithography	-	10	30	Yes	Bluetooth	[193]
	PANI	-		pH	4–7	0.7 over 4 h									
PET/Au	PEDOT	PVC	PVB/NaCl	Na^+^	16–120	-	-	Epidermal patch	Photolithography	-	12	40	-	Bluetooth	[218]
PET/carbon	PANI	-	PVB/NaCl	pH	4–7	3.5	-	Wrist band	Roll-to-roll gravure printing	-	14	51	Yes	Bluetooth	[217]
Paper/carbon	PANI	-	Solid-state Ag/AgCl	pH	4–10	0.5 over one day	-	Epidermal patch	Screen printing	-	-	No	-	No	[219]
Textile/carbon	PANI	-	PVB/NaCl	pH	4.3–8	-	5.0	Bandage	Screen printing	Bending	-	No	-	No	[185]
Tattoo/carbon	PANI	-	Solid-state Ag/AgCl and Nafion/KCl-doped ink	pH	3–7	-	-	Epidermal patch	Screen printing	Bending/stretching	10	50	Yes	No	[220]
PDMS/carbon	CNTs	PVC	Solid-state Ag/AgCl with NaCl-doped PVC and agarose hydrogel	Na^+^	0.1–1000	4.0	-	Epidermal patch	Photolithography	-	-	No	-	No	[221]
Textile-PU/CNTs	CNTs	PU	PVB/NaCl	Na^+^/K^+^	0.1–100	-	-	Textile	Screen printing	Stretching/bending/crumpling	-	No	-	Bluetooth	[177]
Commercial carbon fiber	CNTs	PVC	PVB/NaCl	Na^+^	1–100	0.4 ± 0.3 over 4.5 h	-	Carbon Fiber	Spinning	-	30	80	-	No	[178]
PET/carbon	Carbon	PVC	PVB/NaCl	K^+^	0.1–10	-	-	Eyeglasses	Screen printing	-	10	30	-	Bluetooth	[142]
Tattoo/carbon	Carbon	PVC	PVB/NaCl	Na^+^	0.1–100	2.8	3.0	Epidermal patch	Screen printing	Stretching/bending/poking	15–30	45	-	Bluetooth	[203]
Tattoo/carbon	Carbon	PVC	PVB/NaCl	NH_4_^+^	0.1–100	-	-	Epidermal patch	Screen printing	Stretching/bending	12	36	-	No	[144]
Silicon oxide/Au	AuNDs	PVC	Solid-state PVA/KCl-Ag/AgCl	Na^+^	0.001–100	0.2	8.0	Sweat band	Photolithography	-	10	90	-	No	[140]
PET/Ag-AgCl	silver ink	PU	Solid-state Ag/AgCl with KCl-doped pHEMA	Cl^−^	10–100	3.0	-	Epidermal patch	Screen printing	-	10	5	Yes	No	[222]
Strain redistributed elastic fiber (SSRE-fiber)	AuNPs/CNTs	PVC	PVB/KCl	Na^+^	0.1–100	0.4	-	bandage prototype	deposition	Stretching	-	-	-	No	[223]
PDMS/carbon	rGO	PVC	PVB/NaCl	Na^+^	10–160	9.5	-	Epidermal patch	Screen printing	Stretching, bending, and twisting	20	25	Yes	NFC an-tenna	[224]
		PVC		K^+^	1.0–32	9.5									
	AuNPs	PANI		pH	3–8	5.0									
Paper modified with fluorinated alkyl silane	Graphene	PVC	liquid-junction RE	Na^+^	0.001–100	-	-	Epidermal patch	Spraying and Stencil printing	Bending	-	-	Yes	No	[225]
				K^+^		0.18									
				Cl^−^		-									
				pH	4.0–7.5	-									

Ag/AgCl (silver/silver chloride); Au (gold); AuNDs (gold nanodendrites); AuNPs (gold nanoparticles); AuNS (gold nanosheets); CNTs (carbon nanotubes); DEHP (bis(2-ethylhexyl) phthalate); HMIM-FAP (1-hexyl-3-methylimidazolium tris(pentafluoroethyl)trifluorophosphate); IrOx (iridium oxide); NFC (Near Field Communication); PSS (polystyrene sulfonic acid); pHEMA (poly(2-hydroxyethyl methacrylate)); PMMA (poly(methyl methacrylate)); PMMA-*co*-BMA (poly(methylmethacrylate-*co*-butylmethacrylate)); PU (polyurethane); PVAc (polyvinyl acetate); PVB (polyvinyl butyral); PVC (polyvinyl chloride); RFID (Radio Frequency Identification); TBA-TBB (tetrabutylammonium tetrabutylborate).

### 3.2. Interstitial Fluid

While sweat remains the primary focus in wearable potentiometric sensing devices for assessing human health, interstitial fluid (ISF) is emerging as another crucial biofluid. Its analyte concentrations closely resemble those found in blood samples, providing physiologically relevant data and mitigating the risk of blood component interference and biofouling [226]. Interstitial fluid contains electrolytes such as Na^+^, K^+^, Mg^2+^, and Ca^2+^, along with proteins transported through the capillary endothelium as well as various metabolites, including glucose, cortisol, and ethanol [227,228].

Recent studies have increasingly focused on the development of all-solid-state WPSs for ISF analysis. These devices offer several advantages, including minimal invasiveness and the potential for continuous monitoring. The majority of research efforts have been directed towards enhancing the sensitivity and stability of these sensors, with particular emphasis on the materials used for the electrodes and substrates. For instance, the use of solid-contact materials such as functionalized multiwalled carbon nanotubes (f-MWCNTs) has been shown to significantly enhance the reproducibility and repeatability of these devices [229]. Sensors exhibited a drift as low as 0.35 ± 0.28 mV/h over 13 h. Additionally, innovations in fabrication techniques, such as interference lithography [207], have contributed to more reliable and durable sensor designs.

Despite these advancements, there are gaps that need to be addressed to improve sensing in ISF. One significant challenge is the accurate and reliable extraction of ISF without causing discomfort or damage to the skin. Current microneedle-based systems, while promising, still face issues related to biocompatibility and long-term stability [207,229]. Furthermore, the integration of wireless communication technologies, such as Bluetooth, into these devices remains underexplored. Effective wireless output is crucial for real-time health monitoring and data analysis, yet many studies do not incorporate this feature comprehensively. Lastly, the validation of these sensors under real-world conditions is often lacking. Most studies focus on in vitro or short-duration in vivo tests, leaving a gap in understanding the long-term performance and accuracy of these sensors in practical applications. Table 2 presents an overview of recently published studies on all-solid-state WPSs for ISF analysis.

**Table 2:** Recent Studies on All-Solid-State WPSs for Interstitial Fluid Analysis.

Substrate/Electrode Material	Transducer	ISM Polymer	Reference Electrode	Analyte	Working Range (mM)	Stability (mV h^−1^)	Type of Wearable	Electrode Fabrication	Physical Tests	On-Body Tests Duration (min)	Wireless Output	Ref.
Cotton/carbon	PANI	-	Solid-state Ag/AgCl	pH	3–8	2.5 over 4 h	Cotton Thread	Dip coating	-	1 (In vivo)	Bluetooth	[187]
Steel/carbon	f-MWCNTs	PU	PVB/NaCl	K^+^	0.1–100	0.4	Microneedle	Dip coating and drop casting	Insertion	175 (Ex vivo)	No	[229]
Silicon/porous carbon	3D porous carbon	PVC	Solid-state Ag/AgCl	K^+^	0.01–10	0.2	Microneedle	Interference lithography	-	No	No	[207]
Cotton thread/conductive cotton fiber	SWCNTs	PVC	PVB/NaCl	Li^+^	0.1–63	-	-	dipping	-	-	No	[230]

### 3.3. Other Biofluids

Other biofluids may also be used to monitor human health or provide clinical diagnosis using WPSs, reducing the need for traditional methods. Urine, tears, and saliva are being explored for this purpose [187,193], as shown in Table 3. Challenges are often unique to the type of body fluid. For instance, urine sampling can be time-consuming, typically requiring an entire day for collection to give samples with a representative composition, particularly for tasks like monitoring of creatinine. This necessitates the development of costly wearable devices. Recently, Nyein et al. [193] introduced a WPS based on a flexible printed circuit board made of a disposable material (PET), capable of monitoring Ca^2^^+^ and pH levels in artificial body fluids like sweat, urine, and tears. PANI and PEDOT served as conductive materials in this study.

Saliva is another sample of interest. Lee et al. [231] engineered a wireless, flexible, miniature WPS embedded within a thin electronic platform placed in the oral cavity, enabling long-range monitoring of Na^+^ levels in saliva to manage high blood pressure. The use of mechanically flexible materials ensured compatibility with the oral tissue.

Typical challenges that persist in the application of WPSs for monitoring of human fluids include lifespans that are insufficient for practical healthcare applications. The development of materials and designs that can withstand the dynamic and harsh environments of biofluids remains a critical area of research. Furthermore, the integration of wireless communication technologies and the validation of these devices under real-world conditions are areas that require further exploration.

**Table 3:** Recent publications on WPSs for monitoring other biofluids.

Substrate/Electrode Material	Transducer	ISM Polymer	Reference Electrode	Analyte	Working Range (mM)	Stability (mV h^−1^)	Lifetime (Week)	Type of Wearable	Electrode Fabrication	Physical Tests	Sample	Time to Start On-Body Recording (min)	On-Body Tests Duration (min)	Accuracy Validation	Wireless Output	Ref.
Cotton/CNT yarn	CNT yarn	PVC	-	pH/K^+^/NH_4_^+^	3–11/0.1–100/0.01–10	0.25	8	Cotton yarn	Dip coating	-	Buffer	-	No	-	No	[176]
PET/Ag	PEDOT	PVC	PVB/NaCl	Ca^2+^	0.25–2.0	1.1 over 4 h	-	Sweatband	Photolithography	-	Tears and urine (Artificial)	10	30	Yes	Bluetooth	[193]
	PANI	-		pH	4–7	0.7 over 4 h										
Ecoflex/carbon	PANI	-	Solid-state Ag/AgCl and KCl/Ecoflex	pH	4–10	-	-	Epidermal patch	Laser carbonizing and cutting	Stretching	Buffer	-	No	-	No	[232]
Microporous PDMS/Pd	-	-	Solid-state Ag/AgCl	Na^+^	0.0001–1.0 M	-	1	Intraoral hybrid electronics	Microfabrication Tech.	Bending and stretching	Saliva	-	-	Yes	Bluetooth	[231]

## 4. Conclusions and Future Outlook

The field of wearable potentiometric sensors has made remarkable strides, transitioning from traditional invasive diagnostic tools to sophisticated, non-invasive devices capable of monitoring human biofluids in real time. By replacing conventional electrodes with solid-contact ion-selective electrodes (ISEs), these sensors have enabled significant advancements in miniaturization, reusability, and seamless integration into wearable formats, ensuring user comfort and operational reliability. Progress in solid contacts, including conductive polymers, carbon-based materials, and redox-active compounds, has further enhanced sensor performance, improving their stability, sensitivity, and durability [233]. This review summarizes recent developments in the field, including the sensor mechanisms, design approaches, substrate types, and variety of biofluids targeted by these sensors. Despite these advancements, several challenges remain. Achieving consistent signal stability across varying physiological conditions, preventing biofouling, and addressing potential drifts are critical for ensuring long-term reliability. Environmental factors such as humidity, oxygen, and light, as well as mechanical stress, continue to limit sensor longevity. Moreover, various aspects related to scalability, cost-effective manufacturing, and regulatory compliance must be addressed before these sensors can achieve widespread adoption in clinical and consumer markets.

Looking to the future, the potential of wearable potentiometric sensors lies in addressing these challenges while expanding their capabilities. The development of robust solid-contact materials with enhanced hydrophobicity, high capacitance, and resistance to environmental factors will be pivotal. Nanostructured materials, such as carbon composites and functionalized redox-active substances, hold promise for minimizing signal drift and ensuring reliable long-term performance. Furthermore, integrating advanced wireless technologies, such as Bluetooth, near-field communication (NFC), and cloud-based platforms, will enable real-time health monitoring and seamless data transmission, empowering users and healthcare providers with personalized diagnostic insights. To achieve commercial viability, emphasis must also be placed on scalable and environmentally sustainable manufacturing techniques, such as roll-to-roll, gravure, and inkjet printing. These methods not only reduce production costs but also align with the principles of green electronics by incorporating recyclable and eco-friendly materials. Expanding the scope of wearable devices to detect multiple analytes, including ions, metabolites, and proteins, will open new applications in personalized healthcare, sports performance optimization, and chronic disease management.

User-centric designs that prioritize comfort, flexibility, and ease of integration into daily life are also critical for adoption. Wearable formats such as epidermal patches, textiles, and sweatbands must balance analytical robustness with cost and mechanical durability to withstand the rigors of daily activities. Developing self-calibrating mechanisms and encapsulation strategies will enhance the reliability of long-term monitoring. Collaboration among researchers, industry leaders, and regulatory bodies will be essential to establish standardized performance metrics, ensure safety and data security, and accelerate the translation of these devices into real-world applications. By addressing these challenges, wearable potentiometric sensors can evolve into transformative tools that offer scalable, reliable, and non-invasive solutions for real-time health monitoring, personalized diagnostics, and preventive healthcare, ultimately advancing modern medicine and improving daily well-being.

## Data Availability

Data relevant to the findings of this review are available upon request from the authors.
